# Spatial Evolutionary Characteristics and Influencing Factors of Urban Industrial Carbon Emission in China

**DOI:** 10.3390/ijerph191811227

**Published:** 2022-09-07

**Authors:** Xinyu Zhang, Mufei Shen, Yupeng Luan, Weijia Cui, Xueqin Lin

**Affiliations:** College of Resource Environment and Tourism, Capital Normal University, Beijing 100048, China

**Keywords:** urban industrial carbon emissions, spatial evolution characteristics, influencing factors, spatial Dubin model, China

## Abstract

Climate warming caused by carbon emissions is a hot topic in the international community. Research on urban industrial carbon emissions in China is of great significance for promoting the low-carbon transformation and spatial layout optimization of Chinese industry. Based on ArcGIS spatial analysis, Markov matrix and other methods, this paper calculates and analyzes the temporal and spatial evolution characteristics of industrial carbon emissions in 282 cities in China from 2003 to 2016. Based on the spatial Dubin model, the influencing factors of urban industrial carbon emissions in China and different regions are systematically analyzed. The study shows that (1) China’s urban industrial carbon emissions generally show a trend of first growth and then slow decline. The trend of urban industrial carbon emissions in the western, central, northeastern and eastern regions of China is basically consistent with the overall national trend; (2) In 2003, China’s urban industrial carbon emissions were dominated by low carbon emissions. In 2016, China’s urban industrial carbon emissions were dominated by high carbon emissions, and the spatial trend is gradually decreasing from the eastern region to the central region to the northeast region to the western region; (3) In 2003, the evolution pattern of China’s urban industrial carbon emissions was “low carbon-horizontal expansion” dominated by positive growth, and in 2016, it was “low carbon-vertical expansion” dominated by scale growth; (4) China’s urban industrial carbon emissions have spatial viscosity, and the spatial viscosity decreases with the increase of industrial carbon emissions. (5) In 2004, the relationship between urban industrial carbon emissions and gross industrial output value in China is mainly weak decoupling. In 2016, various types of decoupling regions are more diversified and dispersed, and strong decoupling cities are mainly formed from weak decoupling cities in southwest China and eastern coastal areas; (6) From a national perspective, indicators that are significantly positively correlated with industrial carbon emissions are urban industrial structure, industrial agglomeration level, industrial enterprise scale and urban economic development level, in descending order. Indicators that are significantly negatively correlated with urban industrial carbon emissions are industrial structure and industrial ownership structure, in descending order. Due to the different stages of industrial development and industrial structure in different regions, the influencing factors are also different.

## 1. Introduction

Since the reform and opening up in 1978, China’s industrialization process has been advancing rapidly, and its economic growth has been driven by industries with high energy consumption and high pollution emissions [1]. According to the China Statistical Yearbook, China’s gross industrial product was 162.15 billion yuan in 1978 and 24,786.01 billion yuan in 2016, with a growth rate of 15,185.85%. The total industrial energy consumption increased from 10,008,034 million tons of standard coal to 29,227,596 million tons of standard coal, with a growth rate of 192.04%. A large amount of energy consumption led to an increase in industrial carbon emissions. In 2003, China’s industrial carbon emissions were 2.424 billion tons, accounting for 59.32 percent of the total carbon emissions, and in 2016, 4.695 billion tons, accounting for 52.75 percent of the total carbon emissions. As a party to the United Nations Framework Convention on Climate Change (UNFCCC), China has pledged to reduce its carbon intensity by 60 to 65% by 2030, using 2005 as the base year. In this regard, at the 2020 Climate Ambition Summit, Chinese President Xi Jinping made a clear statement to the world: Guided by the new development concept, China will promote a comprehensive green transformation of economic and social development in the promotion of high-quality development, implement the goal of peaking carbon emissions by 2030, and achieve carbon neutrality by 2060 in order to cope with climate change. In China’s “14th Five-Year Plan for National Economic and Social Development”, it is clearly pointed out that in the next five years, China will continue to increase its support for green technology innovation. For industrial development, it is necessary to promote clean production, develop environmental protection industries, promote the green transformation of key industries and important fields, promote clean, low-carbon, safe and efficient use of energy, and further promote low-carbon transformation in industries and other fields. Facing the arduous task of reducing industrial carbon emissions, China adheres to the development concept of innovation, coordination, green, openness, and sharing. In the future, we will vigorously promote the development of green, low-carbon and circular industries, and take strong actions to deal with climate change.

Based on the reality and urgency of climate change, scholars have paid a lot of attention to carbon emissions. For developed countries and regions, scholars have paid attention to living carbon emissions [2,3]. At the same time, carbon emissions in developing countries and regions such as China and Indonesia mostly focus on industrial carbon emissions and the relationship between economic development and carbon emissions [4,5]. The scales of research are mostly concentrated at national and regional scales [6,7]. For example, Karim Sitara has conducted research on 30 sub-Saharan African (SSA) countries [8]. Industries are mostly concentrated in resource and energy-intensive industries [9,10], scholars who study influencing factors pay more attention to urbanization, economic growth, population size and other influencing factors [11,12,13,14].

The main contributions of this paper are as follows: (1) Research on industrial carbon emissions mainly focuses on the dimensions of countries, regions, and industries. Limited by data availability, there are few studies on industrial carbon emissions at the urban scale. Cities are the basic units of implementing green industrial development policies, so it is necessary to conduct in-depth research on urban scale industrial carbon emission and its influencing factors. (2) The existing researches mainly focus on the influence of regional development conditions such as economic development, industrialization level and technological progress, while less systematic consideration is given to the characteristic factors of Chinese industry. In terms of the selection of influencing factors, this paper, combined with the database of Chinese industrial enterprises, focuses on the factors such as opening up of Chinese industry and technological progress, and in order to influence factors of industrial carbon efficiency has a more comprehensive and scientific scrutiny.

The first part of the study presents the research question. The second part is a literature review, which reviews in detail the analysis of industrial carbon emissions at different spatial scales, related research on carbon emissions in different industries, and related research on factors affecting industrial carbon emissions. The third part introduces the research methods and data sources, and constructs a theoretical analysis framework for the influencing factors of China’s urban industrial carbon emissions. The fourth part describes the empirical results, including the temporal and spatial evolution characteristics of China’s urban industrial carbon emissions from 2003 to 2016, the spatial transfer characteristics of urban industrial carbon emissions, and the empirical analysis results of the influencing factors of industrial carbon emissions in China and different regions. The fifth part summarizes the main conclusions and relevant policy implications.

## 2. Literature Review

At present, the international research on industrial carbon emissions mainly focuses on the following aspects. The first is the measurement and analysis of industrial carbon emissions at different scales. It mainly involves different scales of countries, regions, cities and enterprises. At the national level, industrial carbon emissions mainly discuss the evolution characteristics and laws of total industrial carbon emissions from a macro scale. Feng explored the elasticity of China’s carbon intensity on industrial development, intermediate input coefficient and energy efficiency from 1990 to 2015 [15]. The research on industrial carbon emissions at the regional scale in China is mainly concentrated in the Jiangsu, Hebei and Liaoning provinces with relatively strong industrial foundations [16,17,18]. The issue of industrial carbon emissions in key areas of economic development, such as the Beijing-Tianjin-Hebei and Yangtze River Delta urban agglomerations, has also become a hot issue in the academic circles [19,20]. The research on carbon emissions at the scale of industrial enterprises is mainly carried out through questionnaires and field investigation [21,22].

Some scholars have also measured and analyzed the carbon emissions of different industries. The research mainly focuses on energy-intensive industries such as chemicals, steel, and electricity. HU believes that the steel industry should use carbon-free energy, and safely storing carbon or converting it into harmless substances can greatly reduce carbon emissions [23]. Juan believes that the electricity and ferrous metals industries have the greatest potential for carbon reduction [17].The carbon emission characteristics of emerging industries such as solar photovoltaic and biotechnology have also attracted scholars’ attention [24,25]. Guo believes that photovoltaic power generation systems show good carbon emission reduction effects compared with China’s thermal power generation [26].

The measurement and analysis of industrial carbon emission efficiency is also the research focus of scholars. Scholars usually use the radial analysis method or non-radial analysis method, combined with the ArcGIS spatial analysis method, to measure and analyze the spatial evolution characteristics of industrial carbon emission efficiency at different scales. Karen conducted an environmental efficiency analysis of the Chilean industrial sector using a data envelopment analysis (DEA) model [27]; Sun evaluated the greenhouse gas emission efficiency (GHG efficiency) of 26 industries in China and analyzed its influencing factors using the Stochastic Frontier (SFA) method [28].

Regarding the influencing factors of industrial carbon emissions, from the perspective of international research, scholars generally believe that industrial scale, industrial structure, technological progress, economic development and other factors are the main factors affecting industrial carbon emissions. Regional economic development is the main factor leading to the increase of global carbon emissions. The environmental Kuznets curve (EKC) is a model through the evolution between per capita income and environmental pollution indicators. The relationship between environmental quality and income is an inverted U-shaped relationship [29]. Economic growth affects environmental quality through three ways: scale effect, technology effect and structure effect [30]. Japanese scholar KAYA Yoichi put forward the famous Kaya formula, which posits that the driving force of a country or region’s carbon emissions includes four major factors: population, per capita GDP, energy consumption per unit of GDP, and carbon emissions per unit of energy consumption [31]. Using data from G-6 countries from 1978 to 2014, Nguyen Duc Khuong’s analysis found that the environmental Kuznets curve theory is insufficient to support the conclusion, while economic growth, capital market expansion and trade openness are the main drivers of carbon emissions [32]. In the research on China’s industrial carbon emissions, scholars have actively explored the impact mechanism of economic development and economic scale on China’s industrial carbon emissions [33,34]. At the same time, as a developing country with rapid economic growth, scholars were also concerned about the impact of foreign trade, technological progress, and environmental regulation on China’s industrial carbon emissions [12,35,36]. China’s economic development system based on public ownership has also aroused scholars’ research on the impact of the nationalized economy and land system on industrial carbon emissions [35,36].

This paper uses China’s urban-scale industrial development data and the “China Industrial Enterprise Database” to measure the temporal and spatial evolution characteristics of urban industrial carbon emissions in 282 prefecture-level and above cities in China (excluding Hong Kong, Macao and Taiwan) from 2003 to 2016. The Dubin model analyzes the influencing factors of the spatial evolution of China’s urban scale industrial carbon emissions. This study can provide an important theoretical basis for low-carbon and green transformation and spatial optimization of China’s industry, and provide reference for local governments to formulate scientific and reasonable industrial carbon emission reduction planning and policies.

## 3. Research Methods and Data Sources

### 3.1. Urban Industrial Carbon Emissions Accounting Model

For the measurement of industrial carbon emissions, the currently commonly used measurement methods mainly include the mass balance method [37], the emission factor method, and the actual measurement method. As the most mainstream and core accounting method, the emission factor method is adopted to determine the carbon emission coefficients of various energy sources according to the IPCC carbon emission calculation guidelines [38]. The actual measurement method collects on-site measured data for summarization, and calculates the carbon emissions [39].

In terms of urban-scale carbon emission accounting, commonly used methods include IPCC national Greenhouse gas emission inventory guidelines [40], Oak Ridge National Laboratory methods [31], and National Development and Reform Commission Energy Research Institute published greenhouse gas emission accounting guidelines. The IPCC inventory guide method is widely used by scholars in calculating carbon emissions at the city level, and it is more accurate in fuel division [41,42]. This paper draws on the ideas of other scholars on the calculation of China’s urban-scale industrial carbon emissions, and constructs China’s provincial-level industrial carbon emission coefficients. Referencing IPCC methodology and research by other scholars [43,44], this paper mainly accounts for industrial carbon emissions at city scale from two parts: carbon emissions from fossil fuel combustion and carbon emissions from electricity consumption at industrial terminals. The calculation formula is:

(1) $$E_{t}=E_{1t}+E_{2t}$$

(2) $$E_{1t}=G_{t}\times CG_{t}=\left(\Sigma X_{io}\times A_{io}\times CC_{io}\times CO_{io}\times \left(\frac{44}{12}\right)\right)/G_{i}\times CG_{t}$$

(3) $$E_{2t}=W_{t}\times EFgrid,h$$

In the formula, $E_{t}\;$ is the total carbon emission of *t* city; $E_{1t}$ is the carbon emissions generated by the burning of fossil fuels in each city; $E_{2t}$ is the carbon emission generated by electricity consumption of terminal sectors in each city, $G_{t}$ is the carbon emission coefficient of energy per unit output value of the province where *t* city is located, *o* is the energy category, 12 kinds of energy consumed by industrial fossil fuels are selected, and *i* is the province/city where *t* city is located. $X_{t}$ is the consumption of all kinds of energy, $A_{to}$ is the average low calorific value of the energy *o*. $CC_{i}$ represents the carbon emission factor per unit calorific value equivalent, $CO_{t}$ represents the carbon oxidation rate of energy variety *t*, $G_{i}$ is the total industrial output value of province/city *i* in city *t*, $CG_{t}$ is the total industrial output value of city *t*, $W_{t}$ is the power consumption of industrial terminals in city *t*, EFgrid,h is the average CO_2_ emission factor of regional power grid in city *t*. The low heating values of various energy sources are derived from the China Energy Statistical Yearbook. The power emission factors of each year are selected from the average CO_2_ emission factors of regional power grids released by the Department of Climate Change, National Development and Reform Commission, PRC.

### 3.2. Markov Transition Matrix

The Markov transition matrix reveals the change characteristics of hierarchical composition by measuring the time and state discrete process of things [45]. The study city is discretized into *k* types according to carbon emissions, and the probability distribution of different types in different time periods is calculated to reflect the transfer process of different industrial carbon emission types at the city scale. The formula is as follows:

(4) $$n_{ij}=\frac{m_{ij}}{m_{i}}$$

In the formula, *n_ij_* represents the probability value that the region belonging to type *i* at time *t* is transferred to type *j* at time *t* + 1, *m_ij_* represents the sum of the quantities transferred from type *i* area to type *j* area at time *t* + 1 during the study period, *m_i_* represents the total number of years transferred to type *i* regions during the period.

### 3.3. Decoupling Elasticity Model

The Organization for Economic Cooperation and Development (OECD) proposed the decoupling theory to elaborate the relationship between economic development and environmental pollution. Carbon emission decoupling refers to the relationship between the change of carbon dioxide emissions and the change of industrial output value, and the economic growth elasticity of carbon emissions refers to the decoupling of carbon emissions. According to the OECD decoupling theory and Tapio decoupling model, this paper divides the decoupling status of cities into four categories according to the decoupling elasticity: weak decoupling, strong decoupling, recessive decoupling and negative decoupling [46] (Table 1). The formula for calculating the decoupling elasticity *e* is:

(5) $$e=\frac{\Delta E/E}{\Delta A/A}$$

In the above equation, *E* is industrial carbon emissions (unit: 10.1 million tons), Δ*E* is the added value of industrial carbon emissions (unit: 1010 tons); *A* is the gross industrial output value, and Δ*A* is the growth value of the gross industrial output value, and the unit is one hundred million yuan.

### 3.4. Influencing Factors Analysis Model of Urban Industrial Carbon Emissions

#### 3.4.1. Theoretical Framework Construction

The “Environmental Kuznets hypothesis” proposed by Grossman and Krueger pointed out that changes in the level of urban economic development would lead to a “low-high-low” trend of environmental pollution [29]. The technology effect, scale effect and structure effect accompanied by economic growth are closely related to the change of environmental quality [47]. The “Pollution halo hypothesis” holds that foreign-funded enterprises play a good demonstration effect in the formulation and implementation of technology and environmental protection standards, and the positive spillover of their technology and environmental protection standards has a positive impact on the improvement of national environmental quality. “Porter Hypothesis” explains the relationship between technological innovation and environmental regulation, and believes that appropriate environmental regulation is needed to promote technological innovation of industrial enterprises [30]. Industrial agglomeration theory in the same industry between enterprises of cluster can promote the industry technology spillover, while simultaneous competition can stimulate the industrial enterprise faster and more widely used to create new technologies, namely Marshall externalities [48]. In the socialist market economy system with Chinese characteristics, the important leading role of state-owned economy is constantly emphasized. However, the lack of autonomy of state-owned enterprises, the politicization of regulation, the monopolistic characteristics of the market and the “competition barrier” formed by excessive protection eventually led to low environmental efficiency. Fiscal decentralization is an important institutional basis for the rapid growth of China’s local economy since the reform and opening up in 1978. According to the public choice theory and promotion tournament theory, in order to pursue the rapid growth of local GDP in the short term, local governments will tend to develop polluting industries with high profits and greater contribution to economic growth, and lack motivation for investment in the field of environmental pollution control, which has a slow effect. This will inhibit the upgrading of industrial internal structure, hinder technological innovation, and then lead to the increase of industrial carbon emissions [49,50].

**Figure 1:**
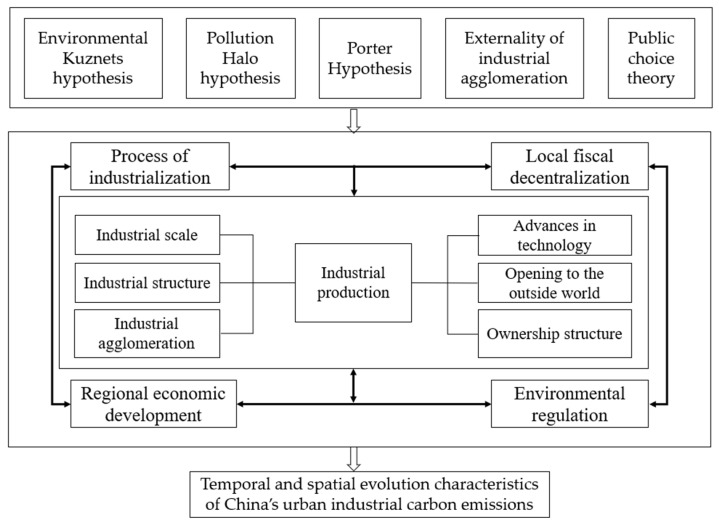
Theoretical framework of influencing factors of China’s urban industrial carbon emissions.

To sum up, this paper summarizes the influencing factors of urban industrial carbon emissions into the following ten factors: economic development level, urban industrial structure, industrial scale, industrial structure, industrial technological progress, industrial opening to the outside world, industrial agglomeration, industrial ownership structure, environmental regulation and local fiscal decentralization. Accordingly, build a theoretical framework of this study (Figure 1).

#### 3.4.2. Model Setting

This article selects the spatial Dubin model, combined with 0–1 matrix relating right weight *W_ij_*, focused on adjacent areas with different degrees of two-way interaction analysis that more accurately measure the influence of different factors on the industrial emissions [51]. It fully reflects that the explained variables in the region are influenced by the mutual influence of adjacent explanatory variables and explained variables, as well as other factors (error terms) not taken into consideration in the city [52]. The formula is as follows:

(6) $$Y_{it}=\alpha_{i}+\rho \overset{N}{\underset{j=1}{\sum }}W_{ij}Y_{jt}+\beta X_{it}+\varphi \overset{N}{\underset{j=1}{\sum }}W_{ij}X_{jt}+\varepsilon_{it}\;$$

(7) $$\begin{array}{ll} lnY_{it}= & a_{0}+a_{1}lnGP+a_{2}lnRIU+a_{3}lnSIE+a_{4}lnRII+a_{5}lnIPI+\\ & a_{6}lnFT+a_{7}lnIC+a_{8}lnEO+a_{9}lnRIE+a_{10}lnIFD \end{array}$$

In the formula, *i* and *j* respectively represent different cities; *t* stands for time; *Y_it_* is urban industrial carbon emissions; *X_it_* is the explanatory variable, where *GP* is the level of economic development, *RIU* is the urban industrial structure, *SIE* is the industrial scale, *RII* is the industrial structure, *IPI* is the industrial technological progress, *FT* is the industrial opening to the outside world, *IC* is the industrial agglomeration, *EO* is the industrial ownership structure, *RIE* is the environmental regulation, *LFD* is the local fiscal decentralization, *i* is the city, *t* represents time, α and $\varepsilon_{it}$ are intercept and random disturbance terms, respectively.

Among them, the l level of economic development is measured by the per capita *GDP*, urban industrial structure is measured by the added value of the second industry of *GDP*, industrial scale is measured by the average size of industrial enterprises, industrial structure is measured by the proportion of the output value of capital and technology intensive industries in the total industrial output value, industry technology progress is measured by the proportion of industrial patent applications in urban patent applications, industry opening to the outside world is measured by the proportion of industrial output value with foreign investment in total industrial output value, industrial agglomeration is measured by urban industrial location quotient, industrial ownership structure is measured by the proportion of output value of state-owned holding industrial enterprises in total industrial output value, environmental regulation is measured by the average weighted summation of urban industrial *SO*_2_ and industrial soot/dust treatment rates, local fiscal decentralization is measured by the proportion of local fiscal expenditures to *GDP*. 

### 3.5. Study Area and Data Sources

**Figure 2:**
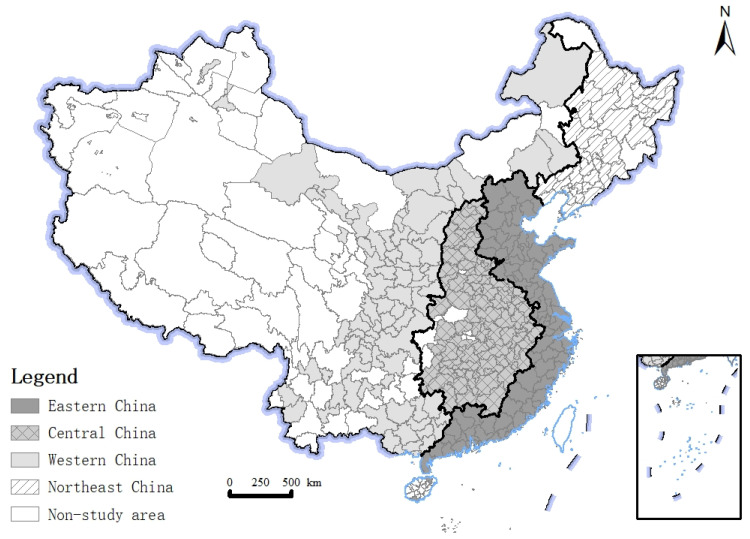
Study area. Note: Produced on the Ministry of Natural Resources Standard Map Service website GS (2019)1823, with no modifications to the base map boundaries.

This paper takes 282 cities at the prefecture level in China as the research object, and reflects the social and economic development of each region in China. Combined with the physical and geographical location conditions, cultural and social conditions, this paper finally divides the region into eastern, central, western and northeast regions according to the “Division Method of East, Central, West and Northeast Regions” published on the official website of the National Bureau of Statistics. The Tibet Autonomous Region, Hong Kong, Macao and Taiwan are not considered (Figure 2).

In this paper, the data of industrial carbon emissions at the urban level and the influencing factors related to regional development from 2003 to 2016 are obtained from “China City Statistical Yearbook” and “China Energy Statistical Yearbook”. Among the influencing factors of industrial carbon emissions, the proportion of output value of capital-intensive industries and the proportion of output value of state-owned holding industrial enterprises are obtained from the China Industrial Enterprise Database, and the data of urban industrial patents are obtained from the matching data between the China Industrial Enterprise Database and patent database. The database of Chinese Industrial Enterprises contains the data of industrial enterprises with an annual main business income of more than 5 million yuan and state-owned industrial enterprises. We processed the data in advance by referring to literature [49,53] and deleted the data: (1) Missing indicators such as gross industrial output value, total assets of enterprises and net value of fixed assets; (2) The actual income is negative or zero; (3) The number of employees is less than 8; (4) The total assets are less than the current assets, the net value of fixed assets and other data that do not conform to accounting principles, excluding the interference of abnormal values in the measurement results.

## 4. Results

### 4.1. Spatial-Temporal Evolution of Urban Industrial Carbon Emissions in China

#### 4.1.1. Time Evolution Characteristics of Urban Industrial Carbon Emissions

**Figure 3:**
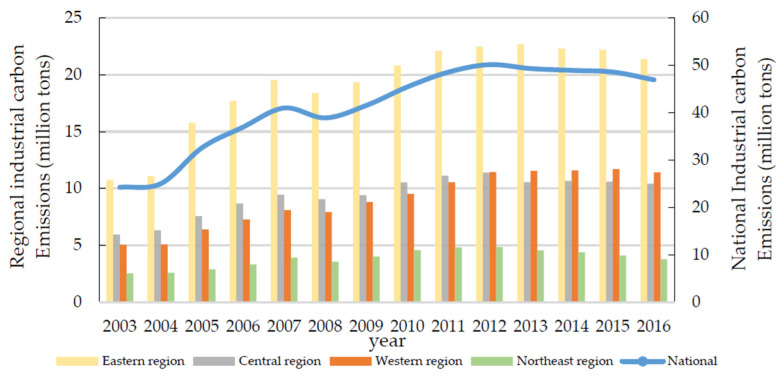
Temporal variation of industrial carbon emissions at city level in China from 2003 to 2016.

From 2003 to 2016, China’s industrial carbon emissions generally increased first and then decreased slowly, which can be divided into three stages. During the period of rapid rise from 2003 to 2007, industrial carbon emissions increased from 2.424 billion tons in 2003 to 4.099 billion tons in 2007, with an average annual growth rate of 4.19%. In 2003, China joined the World Trade Organization (WTO) and further expanded its openings to the outside world. With the rapid development of the industrial economy and the expansion of industrial capacity, industrial carbon emissions have increased rapidly. During the period of steady growth from 2007 to 2012, industrial carbon emissions increased from 4.099 billion tons in 2007 to 5.014 billion tons in 2012, with an average annual growth rate of 1.83%. At this stage, China’s industrial development was greatly affected by the 2008 global financial crisis, resulting industrial capacity shrinkage, and industrial carbon emissions in 2008 decreased by 5.08% compared with 2007. After 2008, driven by domestic demand, the industrial economy resumed growth and industrial carbon emissions kept increasing. In the slow decline stage from 2012 to 2016, industrial carbon emissions decreased from 5014 million tons to 4695 million tons, which is related to the supply-side reform of the economy and China’s economic development entering a new normal during this stage (Figure 3).

The national trend is generally consistent with the trend of industrial carbon emissions in the four regions. The main industrial agglomeration area in China is the eastern region, and the total industrial carbon emissions here is much larger than in other regions. The central region was the second largest industrial carbon emitter before 2012, but it was overtaken by the western region after 2012, ranking third. This is because in the process of industrial transformation and upgrading in the central region, more energy and resource-intensive industries have been transferred to the western region, which has promoted the decline of industrial carbon emissions in the central region and the growth of industrial carbon emissions in the western region. The industrial carbon emissions in the northeast region have always been at the lowest level, which is related to the shrinking scale and slow growth of the industrial economy in the northeast region.

#### 4.1.2. Spatial Evolution Characteristics of Urban Industrial Carbon Emissions

**Figure 4:**
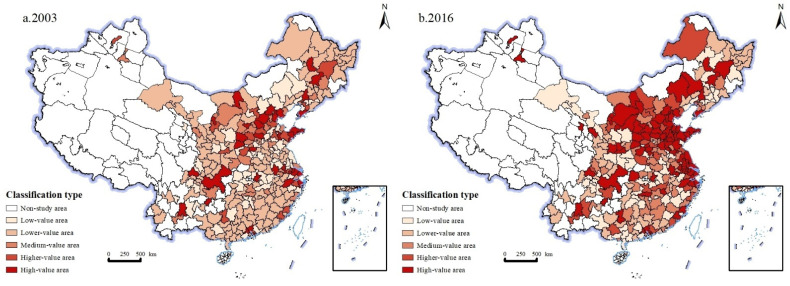
Spatial evolution of urban industrial carbon emissions in 2003 and 2016. (**a**) Spatial evolution of urban industrial carbon emissions in 2003. (**b**) Spatial evolution of urban industrial carbon emissions in 2016.

Urban industrial carbon emissions are divided into five levels: the low value area, the lower value area, median value area, high value area and the higher value area, which are 0.5 times, 1 time, 1.5 times and 2 times of the median, respectively [39]. In 2003, there are 35 higher value areas in China, accounting for 12.4% of the total number of cities, mainly distributed in Guangdong and Shandong Provinces on the east coast and North China, including Guangzhou, Qingdao, Beijing, Chongqing, Shanghai and other cities. These cities are industrial centers for national and even regional development, with large industrial scale and high industrial carbon emissions. There are 19 high value areas, accounting for 6.7% of the total number of cities, mainly distributed in northeast region, North China, eastern coastal region and Xinjiang. There are 32 median value areas, accounting for 11.3% of the total number of cities in China, mostly scattered in Lanzhou, Hengshui, Dongying, Jiaxing and other cities. There are 64 lower value areas, accounting for 22.7% of the total number of cities in China, mainly distributed in the southeast coastal region, northwest region and North China, including Yangzhou, Yan’an, Zhangjiakou and other cities. There are 132 low value areas, accounting for 46.8% of the total number of cities in China, mainly distributed in Anhui Province in the southeast coastal region, Gansu Province in Central China, Jilin and Liaoning Province in the Northeast, including Putian, Jiuquan, Baicheng, Mudanjiang and other cities. In general, in 2003, urban industries in China are dominated by low carbon emissions, with low value areas and low value areas accounting for 69.5% of all cities (Figure 4a). This is related to the low level of industrial development in China, as most cities are in the primary stage of industrialization, and the industrial scale is small.

In 2016, there are 86 higher value areas in China, accounting for 30.4% of the total. Compared with 2003, the number increased significantly, mainly distributed in Shanxi Province in North China and Shandong Province in the northern part of the eastern coastal region, including Taiyuan, Tangshan, Yantai, Qingdao, Tianjin, Shanghai and other cities. These regions are the main clusters of energy and resource-based industries in China, and the large-scale growth of industries has brought about a substantial increase in industrial carbon emissions. There are 27 high value areas, accounting for 9.5% of the total number of cities, mainly distributed in Henan Province in Central China, Hebei Province in North China and Fujian Province in East China, including Chengde, Nanyang, Jincheng, Jinhua and other cities. There are 46 median value areas, accounting for 16.3% of the total number of cities in China, mainly distributed in Henan Province in Central China and Anhui Province in East China, including Puyang, Zhuzhou, Xiangtan, Bengbu and other cities. There are 71 lower value areas, accounting for 25.2% of the total number of cities in China, mainly distributed in Hubei Province in Central China, Sichuan Province in Southwest China and Gansu Province in Northwest China, including Leshan, Panzhihua, Putian, Shantou, Zhangye, Baiyin and other cities. There are 51 low value areas, accounting for 18.1% of the total number of cities in China, mainly distributed in Guangdong and Guangxi in the southeast coastal region, Heilongjiang in the northeast, and Gansu in the northwest, including Shaoguan, Shanwei, Heihe, Hegang, Longnan, Pingliang and other cities (Figure 4). In general, urban industries in China are dominated by high carbon emissions in 2016, and the spatial trend is gradually decreasing from the eastern, central, northeastern and western regions (Figure 4b). The spatial distribution pattern of urban industrial carbon emissions during this period is closely related to the continuous deepening of China’s reform and opening up, the rapid advancement of industrialization, the rapid growth of industrial scale, and the formulation of regional development strategies.

#### 4.1.3. Spatial Evolution Characteristics of Urban Industrial Carbon Emissions Types

**Figure 5:**
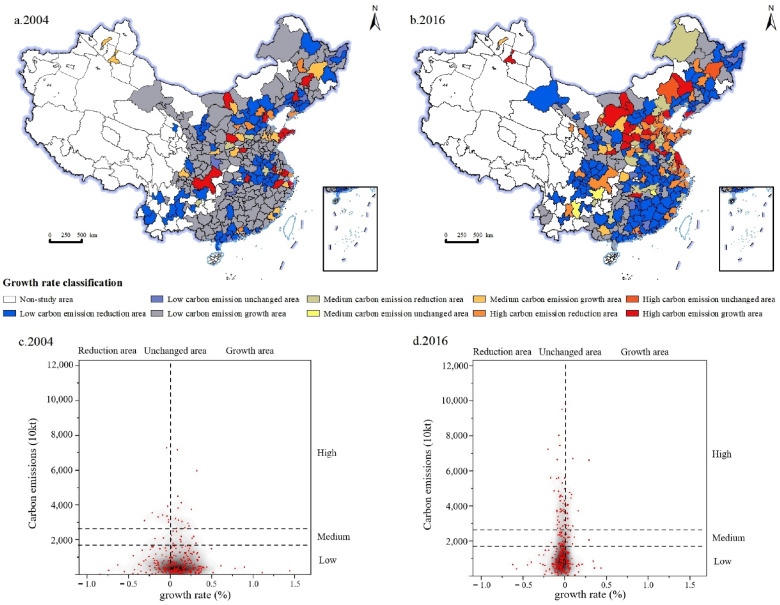
Spatial evolution of urban industrial carbon emissions growth types in 2004 and 2016. (**a**) Spatial evolution of urban industrial carbon emissions growth types in 2004. (**b**) Spatial evolution of urban industrial carbon emissions growth types in 2016. (**c**) Urban industrial carbon emissions and growth rate in 2004. (**d**) Urban industrial carbon emissions and growth rate in 2016.

The growth rate of urban industrial carbon emissions in China during 2003–2004 and 2015–2016 was calculated, with a 0% growth rate as the critical point. By pairwise combination with the aforementioned carbon emission levels, the growth types of industrial carbon emissions can be divided into nine categories: low carbon emission reduction area, unchanged area and growth area, medium carbon emission reduction area, unchanged area and growth area, and high carbon emission reduction area, unchanged area and growth area. In 2004, there are 157 low carbon emission growth areas, accounting for 55.7% of the total in China, mainly distributed in the southeast coastal areas, and the northwest and northeast regions, including Beihai, Chifeng, Huzhou and other cities. There are seven low carbon emission unchanged areas, accounting for 2.5% of the national total, mainly distributed in North China and the Liaoning Province in northeast China, including Ankang, Fushun, Huludao and other cities. There are 71 low carbon emission reduction areas, accounting for 25.2% of the national total, mainly distributed in Guangdong Province in the southeast coastal region, Yunnan Province in the southwest, Hebei Province in North China and Liaoning Province in the northeast, including Putian, Kunming, Baoding and Chengde. There are 16 medium carbon emission growth areas, accounting for 5.7% of the national total, mainly distributed in the southeast coastal areas, including Fuzhou, Jinan, Xiamen and other cities. There are six medium carbon emission reduction areas, accounting for 2.1% of the national total, mainly distributed in Guangdong Province in the southeast coastal region and Shanxi Province in North China, including Dongguan, Foshan and Yuncheng. There are 16 high carbon emission growth areas, accounting for 5.7% of the national total, mainly distributed in the southeast coastal areas, including Hangzhou, Nanjing, Weihai, Yantai and other cities. There are 9 high carbon emission reduction areas, accounting for 3.2% of the country’s total, mainly distributed in the southeast coastal areas and North China, including Guangzhou, Shenzhen, Shanghai, Beijing and other cities (Figure 5a). During this period, most of China was still in the early stage of industrialization, the scale of industrial development was limited, and the total amount of industrial carbon emissions was small. However, with the rapid advancement of urbanization in China, the investment and construction of infrastructure and basic industries has continued to increase, and the transformation and upgrading of traditional consumer goods industries has promoted the rapid development of industries such as heavy chemical industries, and urban industrial carbon emissions have shown the characteristics of rapid growth.

In 2016, there are 123 low carbon emission reduction areas, accounting for 43.6% of the national total, mainly distributed in Guangdong Province in the southeast coastal region, Henan Province in Central China, Sichuan Province in the southwest and northeast China, including Putian, Xinyang, Yibin, Liaoyang and other cities. There are four low carbon emission unchanged areas, accounting for 1.4% of the national total, mainly distributed in Central China, including Hengyang, Jingmen, Shuangyashan and other cities. There are 55 low carbon emission growth areas, accounting for 19.5% of the national total, mainly distributed in the northwest and northeast regions, including Qiqihar, Wuwei, Zhangye and other cities. There are 20 medium carbon emission reduction areas, accounting for 7.1 percent of the national total, mainly distributed in Hebei Province in North China and Henan Province in Central China, including cities such as Chengde, Hengshui and Jiaozuo. There are four medium carbon emission unchanged areas, accounting for 1.4% of the national total, mainly distributed in South China, including Nanchang, Qujing, Xuchang and other cities. There are 9 medium carbon emission growth areas, accounting for 3.2% of the national total, mainly distributed in Central China and North China, including Xianyang, Yichang, Yulin, Jianzhong and other cities. There are 38 high carbon emission reduction areas, accounting for 13.5% of the country’s total, mainly distributed in the southeast coastal areas and North China, including Guangzhou, Shenzhen, Shanghai, Beijing and other cities. There are eight high carbon emission unchanged areas, accounting for 2.8% of the national total, mainly distributed in the southeast coastal areas and North China, including Yantai, Zibo, Chifeng, Handan and other cities. There are 21 high carbon emission growth areas, accounting for 7.4% of the national total, mainly in Shaanxi province in North China, including Taiyuan, Xi’an, Yuncheng, Hohhot and other cities (Figure 5b). In 2001, China officially joined the World Trade Organization, and the country’s degree of opening to the outside world continued to improve. China’s domestic industrial enterprises are facing globalized market competition, forcing enterprises to transform and upgrade. The report of the 16th National Congress of the Communist Party of China pointed out that it is necessary to insist on driving industrialization with informatization and promoting informatization with industrialization. A new type of industrialization road with high scientific and technological content, good economic benefits, low resource consumption, less environmental pollution, and full use of human resources advantages. With the implementation of the new industrialization strategy, China’s industrial structure has been continuously optimized, and the level of energy utilization has been continuously improved. Therefore, in 2016, from a national perspective, the overall industrial carbon emissions showed a change in the characteristics of reduction and low growth.

The evolution pattern of urban industrial carbon emission types in China in 2004 can be summarized as the “low-carbon horizontal expansion” type dominated by rate growth, that is, forming a core dominated by low-carbon emissions and showing an obvious positive growth rate of carbon emissions (Figure 5c). The evolution pattern of industrial carbon emission types in 2016 can be summarized as the “low-carbon longitudinal expansion” type dominated by scale change, that is, the evolution characteristics mainly characterized by the scale growth of carbon emissions, accompanied by a low growth rate of carbon emissions (Figure 5d).

#### 4.1.4. Spatial Transfer Characteristics of Urban Industrial Carbon Emissions

We calculated the Markov transition probability matrix of China’s urban industrial carbon emission grade types in 2003–2007, 2007–2012, and 2012–2016 (Table 2). The low value area, the lower value area, the median value area, the high value area and the higher value area of industrial carbon emissions correspond to *k* = 1, 2, 3, 4, 5, and the larger *k* refers to the larger industrial carbon emissions. The movement from the low value area to the high value area is defined as upward movement, and, vice versa, is defined as downward movement. It can be seen that in the time series, the value on the diagonal is the probability of no change in the area of industrial carbon emission type, and the value on the off-diagonal is the probability of its transformation. The spatial transfer characteristics of industrial carbon emissions are as follows: (1) In different development stages and different levels of industrial carbon emissions, the probability of urban non-transfer is the highest among all the transfer probabilities, industrial carbon emissions have space viscosity, and the industrial carbon emissions increase; this kind of space viscosity decreases continuously, but in industrial carbon emissions, which are high value areas, space and viscosity rise significantly. This is consistent with the research conclusions of Wang, who believes that China’s urban carbon emissions are stable, and there is a phenomenon of “club convergence”, which makes it difficult to achieve leapfrog development in adjacent years [54]. The spatial viscosity of cities with different levels of industrial carbon emission from 2003 to 2007 is lower than that from 2007 to 2012 and 2012 to 2016. (2) Except for the high value area level, the probability of cities with different industrial carbon emission levels transferring to cities with high industrial carbon emission levels is much higher than that of cities with low industrial carbon emission levels, and the transfer is mainly to neighboring cities with high carbon emission levels. From 2003 to 2007, the probability of transfer from *k* to *k* + 1 increased successively. From 2007 to 2012 and 2012 to 2016, except for the transfer type from low-value area to low-value area, the probability values of other transfer types showed a decreasing trend. This characteristic of change is related to the resource dependence of urban development. For example, Taiyuan City in Shaanxi Province was developed on the basis of abundant coal resources. Resource-based industries account for a high proportion of urban economic development, and economic development is highly dependent on resources, making urban transformation and development difficult, and more inclined to rely on the original development path to continue development, which will bring more industrial carbon emissions.

#### 4.1.5. Evolution Characteristics of Decoupling Relationship between Urban Industrial Carbon Emissions and Industrial Growth Rate

**Figure 6:**
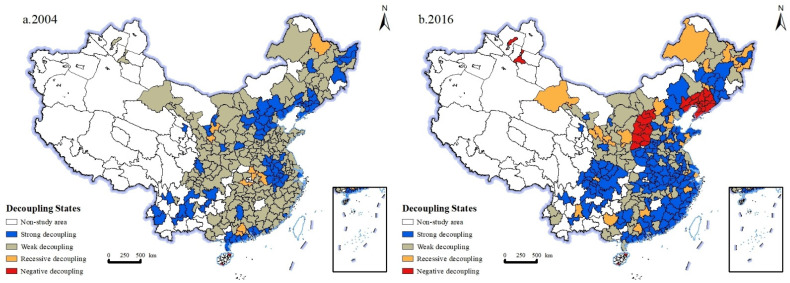
Distribution map of decoupling elasticity of China’s urban industrial carbon emissions in 2004 and 2016. (**a**) Distribution map of decoupling elasticity of China’s urban industrial carbon emissions in 2004. (**b**) Distribution map of decoupling elasticity of China’s urban industrial carbon emissions in 2016.

According to Formula (5), the decoupling relationship between urban industrial carbon emissions and industrial added value in China in 2004 and 2016 is calculated and spatially visualized (Figure 6). It can be seen that in 2004, the relationship between industrial carbon emissions and industrial added value in Chinese cities is mainly weak decoupling, with 187 cities accounting for 66.31% of all cities, mainly distributed in northeast China, Northwest China, Central China and southeast coastal areas, including Tianjin, Shenyang, Nanjing, Wuhan, Lanzhou and other cities. Followed by strong decoupling cities, there are 78 cities in total, accounting for 27.66% of all cities, mainly distributed in the Bohai Rim region, including Beijing, Shijiazhuang, Taiyuan and other cities. There are 10 recessive decoupling cities, accounting for 2.82% of the total cities, mainly distributed in Central China, including Huanggang, Jingzhou, Guyuan and other cities. Weak decoupling indicates that both urban industrial carbon emissions and the economy are growing positively, but the growth rate of industrial carbon emissions is less than that of economic growth. The rapid advancement of the overall industrialization during this period made industrial carbon emissions and economic growth show a synergistic growth trend. While the use of resources and energy has increased significantly, energy efficiency has also continued to improve, which makes the relationship between urban industrial carbon emissions and industrial output value show a weak decoupling feature.

In 2016, the relationship between industrial carbon emissions and industrial added value in Chinese cities is dominated by strong decoupling, with a total of 145 cities, accounting for 51.42% of all cities, mainly distributed in northeast China, Southwest China, Central China and southeast coastal areas, including Beijing, Changchun, Zhengzhou, Guangzhou and other cities. There are 73 weak decoupling cities, accounting for 25.89% of the total cities, mainly distributed in northeast China, North China and northwest China, including Qiqihar, Tangshan, Hohhot, Xi’an and other cities. There are 30 recessive decoupling cities, accounting for 10.64% of the total cities, mainly distributed in Central China, including Huanggang, Jingzhou, Guyuan and other cities. There are 29 negative decoupling cities, accounting for 10.28% of the total cities, mainly distributed in the northwest and northeast regions, including Daqing, Lanzhou, Jiuquan, Guyuan and other cities. Strong decoupling means economic growth and environmental pressure reduction. In the context of ecological civilization construction, in this period, China achieved significant emission reduction and maintained economic growth. This is consistent with the findings of Sun [28].

### 4.2. Overall Influencing Factors of Urban Industrial Carbon Emissions

According to the aforementioned Equation (7) setting, a spatial Durbin model regression is conducted in STATA 16.0 to analyze the influencing factors of industrial carbon emissions in most of China’s regions. The individual and time double fixed effects are uniformly selected, and the results are shown in Table 3 below.

#### 4.2.1. Overall Influencing Factors of Urban Industrial Carbon Emissions

From a national perspective, the indicators that are significantly positively correlated with industrial carbon emissions are, from large to small, urban industrial structure, industrial agglomeration, industrial scale, and economic development level. The higher the proportion of the output value of the secondary industry, the higher the level of industrial scale development, and accordingly the consumption scale of coal, crude oil, natural gas and other fuels will be expanded in the production process, resulting in more carbon emissions [55]. An increased level of industrial agglomeration represents the expansion of industrial scale in a certain spatial area, which in turn brings more carbon emissions from industrial production [56]. The larger the industrial enterprise, the larger the scale of production, and therefore it exhibits a synergistic change effect with the growth of industrial carbon emissions. In China, industrial development is the dominant force driving economic growth, and higher levels of economic development often mean larger industrial production and a higher share of industrial output. Therefore, economic growth and industrial carbon emissions tend to change synergistically.

The indicators that are significantly and negatively correlated with urban industrial carbon emissions are industrial structure and industrial ownership structure. The larger the output value of capital and technology-intensive industries in the industrial structure, the higher the level of technology, automation, and mechanization of local industries. State-owned holding enterprises are generally larger in scale and have a greater ability to carry out R&D investment in green production technologies. Therefore, changes in these two factors will help reduce industrial carbon emissions.

In the exogenous interaction effects of factors affecting industrial carbon emissions in Chinese cities, local fiscal decentralization and the improvement of industrial structure will increase industrial carbon emissions in adjacent areas. This is because urban construction and industrial development promote industrial economic development on the basis of improving the investment environment, which will attract more related industrial enterprises to gather in adjacent areas and promote the increase of industrial carbon emissions in adjacent areas.

The expansion of urban industrial structure, industrial technology progress, economic development level, industrial scale, and industrial agglomeration will decrease the industrial carbon emissions of neighboring areas. The improvement of regional industrialization levels, the increase of industrial patents, the rise of economic development level, the size of industrial enterprises and the level of industrial agglomeration all contribute to the positive radiation effect on the technology and management of industrial enterprises in neighboring areas which can foster industrial transformation and upgrading, increase technology research and environmental protection expenditure, and thus reduce industrial carbon emissions in neighboring areas.

Li et al. analyzed the influencing factors of carbon emissions in 39 industrial sectors in China, and found that the industrial structure was significantly positively correlated with carbon emissions [57]. Pan believes that there is a significant positive correlation between economic development and China’s industrial carbon emissions [58]. The above conclusions are consistent with the point of view of this paper. Han believes that technological progress is significantly negatively correlated with China’s industrial carbon emissions [59]. However, the influencing factors of technological progress in this paper have no significant impact on China’s industrial carbon emissions. This may be due to the limited access to indicators; we did not obtain indicators of technological progress at the industrial level, but rather at the urban level, including technological progress in all aspects of the city. Therefore, it does not show a significant correlation with urban industrial carbon emissions.

#### 4.2.2. Influencing Factors of Urban Industrial Carbon Emissions in Different Regions

The positive influences on industrial carbon emissions in the eastern region are consistent with the national level. The indicators that are obviously and directly related to industrial carbon emissions are urban industrial structure, industrial agglomeration level, urban economic development level, and industrial enterprise scale, in descending order. The indices that are strongly and adversely correlated with industrial carbon emissions are industrial structure and level of industrial openness. With the deepening of China’s reform and its opening up, foreign exchanges will inevitably accelerate the import of high precision technology and the optimization of industrial structure transformation, the proportion of high-carbon industries decline or low-carbon development, in order to achieve a win-win situation of economic and ecological benefits. Among the exogenous interaction effects of the factors influencing industrial carbon emissions in the eastern region, the level of industrial openness to the outside world, the structure of industrial ownership, and the decentralization of local finance from large to small show significant positive correlations with industrial carbon emissions. Meanwhile, urban industrial structure, industrial agglomeration level, and industrial technology progress are significantly and negatively correlated with industrial carbon emissions of neighboring cities. The high level of industrialization in the eastern region generates technology and management spillover in the process of local industrial transformation and upgrading and agglomeration, which promotes the transformation and upgrading of regional industrial structure and thus reduces carbon emissions in neighboring regions.

The indicators that are clearly and visibly correlated with industrial carbon emissions in the central region are industrial agglomeration, industrial structure, economic development level, and industrial scale, in descending order. Industrial openness and industrial ownership structure are negatively correlated with industrial carbon emissions. Foreign-invested and state-owned enterprises tend to have higher pollution control technology, which facilitates green technology innovation activities and makes industrial carbon emission reduction. Among the exogenous interaction effects of factors affecting industrial carbon emissions in the central region, the factors that are significantly negatively correlated with industrial carbon emissions are the level of industrial agglomeration and the level of urban economic development, in descending order. The large-scale development and economic growth of urban industries in the central region will further promote the agglomeration of related industries in the region, thereby reducing the pressure on industrial carbon emissions in adjacent regions.

The indicators that are positively correlated with industrial carbon emissions in the western region are industrial structure, industrial scale, industrial agglomeration, and economic development level, in descending order. The only indicator that is negatively correlated with industrial carbon emissions is the industrial ownership structure. Among the exogenous interaction effects of factors influencing industrial carbon emissions in the western region, the level of industrial openness to the outside world, local fiscal decentralization and industrial carbon emissions showed a significant positive correlation. For the western region, the improvement of the level of industrial openness to the outside world and the construction of a perfect urban investment environment will accelerate the level and scale of industrial agglomeration, which will lead to the development of industrial scale in the surrounding areas and thus promote the increase of industrial carbon emissions in neighboring areas. However, the correlation coefficient between these two factors is small due to the development of urban industry in the western region being subject to the role of national policy regulation, so the spillover effect and radiation-driven impact on neighboring areas is not prominent [60]. The factors that show decreasing correlation with industrial carbon emissions are industrial agglomeration levels and urban economic development levels, in descending order.

The indicators that are significantly positively correlated with industrial carbon emissions in Northeast China are urban industrial structure, industrial agglomeration, economic development level, and industrial scale, in descending order. The indicator that is negatively correlated with industrial carbon emissions is industrial ownership structure. Among the exogenous interaction effects of factors influencing industrial carbon emissions in Northeast China, the factors that are significantly and positively related to industrial carbon emissions in descending order are local fiscal decentralization and industrial structure. The factors that are significantly negatively correlated with industrial carbon emissions are economic development level, industrial agglomeration, and industrial ownership structure, in descending order.

In different regions, the influencing factors of urban industrial carbon emissions are heterogeneous. In different regions, the level of economic development and industrial structure have a significant positive impact on urban industrial carbon emissions. However, due to the different characteristics of industrial structure and economic development in different regions, the impact of these two factors on urban industrial carbon emissions does not show regular changes. There are also some factors that show regular changes in the impact of urban industrial carbon emissions in regions with different economic development levels. For example, in a region with a higher level of economic development, the level of industrial agglomeration has a greater positive impact on industrial carbon emissions, and the scale of industrial enterprises has a smaller positive impact on industrial carbon emissions. Capital-intensive industries and foreign investment levels have a greater negative impact on industrial carbon emissions. The proportion of state-owned enterprises has less negative impact on industrial carbon emissions. In addition, except for the central region, industrial technological progress has no significant effect on urban industrial carbon emissions in China and different regions, and environmental regulation and local fiscal decentralization have no significant effect on industrial carbon emissions in China and different regions. The heterogeneity and regular changes of these influencing factors can guide China and different regions to formulate and improve policies that can effectively guide the low-carbon and green development of industries.

## 5. Conclusions

Based on ArcGIS spatial analysis, Markov matrix, spatial Dubin model and other methods, this paper calculates and analyzes the spatial-temporal evolution characteristics and influencing factors of urban industrial carbon emissions in China from 2003 to 2016. The results show that:

(1) From 2003 to 2016, China’s urban industrial carbon emissions showed a general trend of increasing first and then slowly decreasing, which can be divided into three stages. The trend of industrial carbon emissions in eastern, central, western and northeast China is generally consistent with the overall national trend.
(2) In 2003, China’s urban industrial carbon emissions are mainly low carbon emissions, and in 2016, the industrial carbon emissions are mainly high carbon emissions. Spatially, the industrial carbon emissions showed a trend of gradual decrease from the eastern region to the central region to the northeast region to the western region.
(3) In 2004, China’s urban industrial carbon emissions are mainly in low carbon emission growth areas, and in 2016, China’s urban industrial carbon emissions are mainly in low carbon emission reduction areas, which is in the steady evolution process of carbon emissions to low value areas. In 2004, the evolution pattern of urban industrial carbon emissions in China is “low-carbon horizontal expansion” led by positive growth, and in 2016, the evolution pattern of industrial carbon emissions is “low-carbon vertical expansion” led by scale growth.
(4) Urban industrial carbon emissions have spatial viscosity, and the spatial viscosity decreases with the increase of industrial carbon emissions. Except for the high value area level, the probability of cities with different urban industrial carbon emission levels transferring to cities with high industrial carbon emission levels is much higher than that of cities with low industrial carbon emission levels, and the transfer is mainly to neighboring cities with high carbon emission levels.
(5) In 2004, the relationship between urban industrial carbon emissions and gross industrial output value in China was dominated by weak decoupling. In 2016, the relationship between urban industrial carbon emissions and gross industrial output value in China was dominated by strong decoupling, and the strong decoupling cities are mostly transformed from the weak decoupling cities in southwest China and eastern coastal areas. Each type of decoupling area is more diverse and more dispersed.
(6) From the national point of view, industrial structure, industrial agglomeration, industrial scale and economic development level are the indicators significantly positively correlated with industrial carbon emissions. Industrial structure and industrial ownership structure are the indexes that are significantly negatively correlated with urban industrial carbon emissions from large to small. Due to the different stages of industrial development and the different characteristics of industrial structure, the influencing factors are also different in different regions.

For China, it is necessary to accelerate the low-carbon development of traditional industries, promote the process of clean production transformation, and build a clean and low-carbon energy system. Enterprises and large enterprise groups should play the leading role, implement a number of major projects with outstanding carbon reduction effects and strong driving force in major carbon emission industries. For industries in energy-rich cities, under the premise of strictly protecting the ecological environment, they should improve the green supply capacity of energy resources, accelerate the construction of green and low-carbon development highlands, focusing on cities in economically developed regions such as the Beijing-Tianjin-Hebei, Yangtze River Delta, Guangdong-Hong Kong-Macao Greater Bay Area and other regions. China should coordinate the adjustment of regional industrial structure, promote industrial transfer of traditional industries, cross-regional coordination of industrial chains, efficient industrial aggregation, and promote the optimal allocation of regional energy resources. Improve the industrial technology innovation system, and strengthen the supporting role of technological innovation in the green and low-carbon transformation of the industry.

The eastern region has a relatively high level of industrialization. In the future, it should focus on building strategic emerging industries with low energy and resource consumption, less environmental pollution, and high added value and strong market demand, so as to drive the transformation and upgrading of the regional industrial structure and the green and low-carbon development of the economy and society. It is necessary to intensify the innovation of green technology and cultivate and build a number of technology innovation centers in the green and low-carbon fields through the implementation of green technology innovation tackling actions. Eastern region should strengthen international exchanges and cooperation in green and low-carbon technologies, and accelerate breakthroughs in the engineering and industrialization of green and low-carbon technologies. The central region should continue to open up to the outside world and expand the development space for industry. Central region should continue to deepen reforms to provide a favorable market environment for industrial enterprises to develop in the central region. The western region should continuously optimize the development environment of state-owned enterprises, and strengthen investment in key areas such as innovation platforms, investment and financing platforms. The Northeast region should actively promote the transformation and development of resource-based regions and the adjustment and transformation of old industrial cities, accelerate the low-carbon development of traditional industries such as iron and steel, and promote the green development of key industries such as the chemical and coal industries. It should also transform and upgrade traditional advantageous industrial industries, cultivate and expand emerging industries, and focus on improving innovation support capabilities.

Based on the construction of the provincial scale industrial carbon emission coefficients, this paper calculated and analyzed the urban scale industrial carbon emissions in China. However, the downscaling study will inevitably produce errors. With the support of accurate data available in the future, we will continue to conduct in-depth research on this issue. In addition, this paper only analyzes the overall urban industrial carbon emissions. In-depth research on the carbon emissions of different industrial sectors within the industry is of more practical significance for promoting the low-carbon and green development of the industry and realizing the “carbon peaking and carbon neutrality” goal, which will also be the focus of the next study.

## Figures and Tables

**Table 1:** Decoupling type partition table.

Decoupling Type		Industrial Carbon Emissions Increment	Total Industrial Output Increment	Decoupling Elasticity
Decoupling	Strong decoupling	<0	>0	<0
	Weak decoupling	>0	>0	0 < *e* < 1
Negative decoupling		>0	>0	*e* > 1
		>0	<0	*e* < 0
		<0	<0	0 < *e* < 1
Recessive decoupling		<0	<0	*e* > 1

**Table 2:** Markov matrix for urban industrial carbon emissions of China in 2003–2016.

Period	t/t + 1	Low	Lower	Median	High	Higher
2003–2007	Low	0.7949	0.1399	0.0652	0.0000	0.0000
	Lower	0.0457	0.6981	0.2355	0.0207	0.0000
	Median	0.0000	0.0369	0.6797	0.2462	0.0372
	High	0.0000	0.0000	0.0763	0.5974	0.3263
	Higher	0.0000	0.0000	0.0193	0.0445	0.7362
2007–2012	Low	0.8307	0.1693	0.0000	0.0000	0.0000
	Lower	0.0521	0.7978	0.1377	0.0124	0.0000
	Median	0.0000	0.084	0.7571	0.1552	0.0037
	High	0.0000	0.0057	0.0844	0.6985	0.2114
	Higher	0.0000	0.0153	0.0282	0.0891	0.8674
2012–2016	Low	0.7962	0.1596	0.0442	0.0000	0.0000
	Lower	0.0829	0.7771	0.1214	0.0186	0.0000
	Median	0.0042	0.0983	0.7502	0.1372	0.0101
	High	0.0000	0.0104	0.132	0.7124	0.1452
	Higher	0.0000	0.0092	0.0352	0.1285	0.8271

**Table 3:** Results of the SDM model on the influencing factors of China’s urban industrial carbon emissions from 2003 to 2016.

Variable	National		Eastern Region		Central Region		Western Region		Northeast Region	
	Coefficient	T	Coefficient	T	Coefficient	T	Coefficient	T	Coefficient	T
ln*GP*	0.2530 ***	(13.70)	0.2640 ***	(9.35)	0.3170 ***	(10.34)	0.1450 ***	(4.86)	0.5610 ***	(8.15)
ln*RIU*	1.810 ***	(23.54)	1.440 ***	(11.24)	0.5550 ***	(4.20)	2.0250 ***	(15.85)	1.3190 ***	(5.85)
ln*SIE*	0.2650 ***	(19.87)	0.1180 ***	(4.98)	0.1850 ***	(8.79)	0.3310 ***	(13.60)	0.1080 ***	(3.41)
ln*RII*	−0.2040 ***	(−4.80)	−0.5580 ***	(−6.03)	0.0268	(0.40)	−0.0068	(−0.10)	−0.1400	(−1.56)
ln*IPI*	0.0414	(1.65)	0.0512	(1.34)	0.0932 **	(2.94)	0.0617	(1.30)	−0.0437	(−0.53)
ln*FT*	−0.0903	(−1.29)	−0.1910 *	(−2.00)	−0.4360 ***	(−3.80)	0.1440	(0.96)	−0.1780	(−1.38)
ln*IC*	0.3360 ***	(27.08)	0.7080 ***	(29.58)	0.7160 ***	(22.58)	0.2050 ***	(11.25)	0.5700 ***	(12.61)
ln*EO*	−0.1140 ***	(−4.33)	−0.0166	(−0.29)	−0.0917 *	(−2.31)	−0.1040 *	(−2.19)	−0.1440 **	(−3.03)
ln*RIE*	0.0235	(0.94)	0.0125	(0.35)	0.0837 *	(2.47)	0.0496	(1.11)	−0.0581	(−0.74)
ln*LFD*	0.0180	(0.34)	0.0104	(0.14)	0.3330	(1.82)	0.0302	(0.41)	−0.2480	(−1.21)
W*ln*GP*	−0.1730 ***	(−6.43)	−0.0579	(−1.34)	−0.4670 ***	(−8.22)	−0.0509	(−1.18)	−1.2810 ***	(−9.31)
W*ln*RIU*	−1.7360 ***	(−15.43)	−0.8390 ***	(−4.39)	−0.1270	(−0.61)	−1.5180 ***	(−7.54)	0.7780	(1.64)
W*ln*SIE*	−0.1620 ***	(−7.78)	0.0340	(0.95)	−0.0341	(−1.10)	−0.2020 ***	(−5.47)	0.0503	(0.84)
W*ln*RII*	0.2550 ***	(3.38)	0.0310	(0.18)	0.0456	(0.35)	0.1300	(1.03)	0.6470 ***	(3.56)
W*ln*IPI*	−0.3290 ***	(−7.00)	−0.1540 *	(−2.12)	−0.1490 *	(−2.20)	−0.2230 **	(−2.62)	0.0795	(0.42)
W*ln*FT*	0.2740	(1.92)	0.1610	(0.89)	−0.4420	(−1.51)	0.7230 **	(2.59)	0.6270 *	(2.10)
W*ln*IC*	−0.1080 ***	(−7.07)	−0.4750 ***	(−11.44)	−0.5660 ***	(−9.91)	−0.0425 *	(−2.07)	−0.5970 ***	(−6.14)
W*ln*EO*	0.0296	(0.64)	0.1440	(1.24)	−0.1090	(−1.45)	−0.1440	(−1.69)	−0.3850 ***	(−4.07)
W*ln*RIE*	−0.0851	(−1.79)	−0.0114	(−0.17)	−0.2010 **	(−2.68)	−0.0985	(−1.18)	0.1900	(1.41)
W*ln*LFD*	0.3790 ***	(3.43)	0.1290	(0.83)	0.8210 *	(2.25)	0.3860 *	(2.17)	1.1180 ***	(3.44)
Spatial_rho	0.4890 ***	(29.09)	0.4460 ***	(13.52)	0.5470 ***	(18.97)	0.4880 ***	(17.61)	0.3510 ***	(6.53)
Variance sigma^2^_e	0.0228 ***	(43.61)	0.0110 ***	(24.07)	0.0101 ***	(22.92)	0.0282 ***	(23.43)	0.0149 ***	(15.24)
r^2^	0.238		0.496		0.289		0.422		0.0574	

Note: ***, **, and * denote significance levels of 1%, 5%, and 10%, respectively.

## Data Availability

Not applicable.
